# Nonoperative Management in Intact Burst Fracture Patient With Thoracolumbar Injury Classification and Severity Score of 5: A Case Report

**DOI:** 10.7759/cureus.29492

**Published:** 2022-09-23

**Authors:** Gersham J Rainone, Yash Patel, Cody Woodhouse, Ryan Sauber, Alexander Yu

**Affiliations:** 1 Medicine, Drexel University College of Medicine, Philadelphia, USA; 2 Neurosurgery, Allegheny General Hospital, Pittsburgh, USA; 3 Orthopaedic Surgery, Allegheny Health Network, Pittsburgh, USA

**Keywords:** thoracolumbar injury classification and severity (tlics), thoracolumbar fracture, ligamentous injury, non-operative management, burst fracture

## Abstract

Thoracolumbar fractures are a common consequence of trauma, often a result of motor vehicle accidents or falls. Burst fractures are a morphology of thoracolumbar fracture in which compressive force causes retropulsion of the posterior elements of the vertebral body, potentially leading to neurological deficits. The Thoracolumbar Injury Classification and Severity (TLICS) score is a decision-making tool to help surgeons decide between nonoperative and operative management. For assigned scores of 4, management is at the discretion of the surgeon, and for scores ≥ 5, operative treatment is recommended. Burst fracture patients that are neurologically intact are given a score of 5 if there is a posterior ligamentous complex (PLC) injury and are recommended to undergo operative management. Here we present a neurologically intact patient with an L4 burst fracture with PLC injury that was managed conservatively and demonstrated successful clinical, functional, and radiographic recovery.

## Introduction

Thoracolumbar fractures of T10-L2 are the most common fractures of the spinal column. There are about 25,000 burst fractures annually in the US and 4.4% of all traumas had thoracolumbar fractures [1]. Management of thoracolumbar fractures in neurologically intact patients is controversial [2]. The Spine Trauma Study Group proposed the thoracolumbar injury classification and severity (TLICS) score to help guide management based on the morphology of injury, neurologic status, and integrity of the posterior ligamentous complex (PLC) [3,4]. In each category, the patient is assigned a numeric score, and the sum of these quantitative measurements is used as guidance for the management of the patient. For a TLICS score of ≤ 3 conservative management is recommended, for a score of 4, the management decision is based on the judgment of the surgeon, and for a score of ≥ 5, operative management is recommended. Although these scores have been useful for decisions in management, it is not uncommon that patients with a TLICS score of ≤ 3 have required operative management [5,6]. There has also been one case reported in the literature where a patient with a TLICS score of ≥ 5 was successfully managed without surgical stabilization [7]. We add an additional case to the literature of a neurologically intact patient with a burst fracture and PLC injury that was managed without surgical stabilization and was successful in their recovery from injury.

## Case presentation

**Figure 1:**
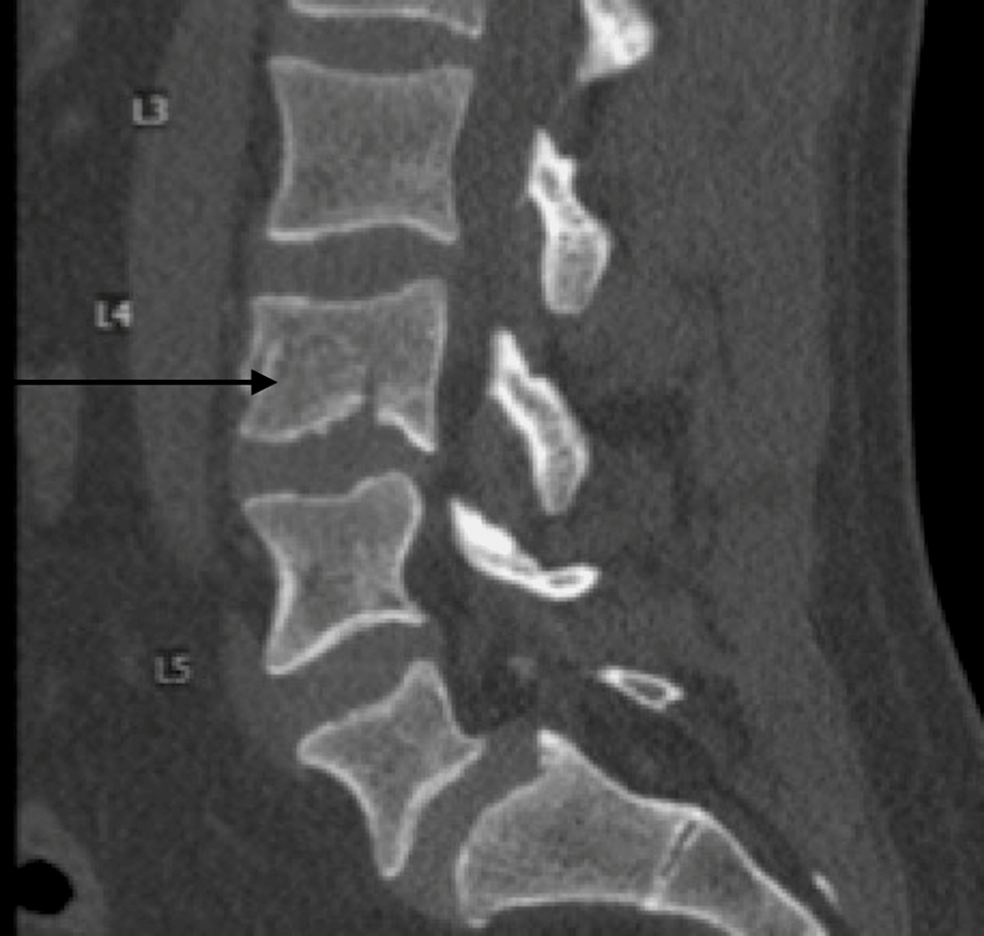
L4 burst fracture

**Figure 2:**
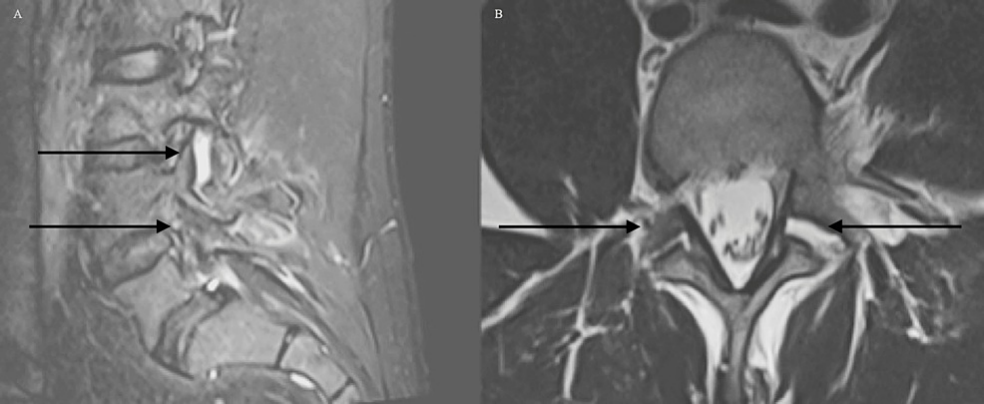
MRI of the lumbar spine A) Sagittal STIR sequence demonstrating widening of the left facet with evidence of facet capsule injury; B) axial T2 sequence demonstrating widening and edema of bilateral L4/5 facet joints left worse than right STIR: short tau inversion recovery

**Figure 3:**
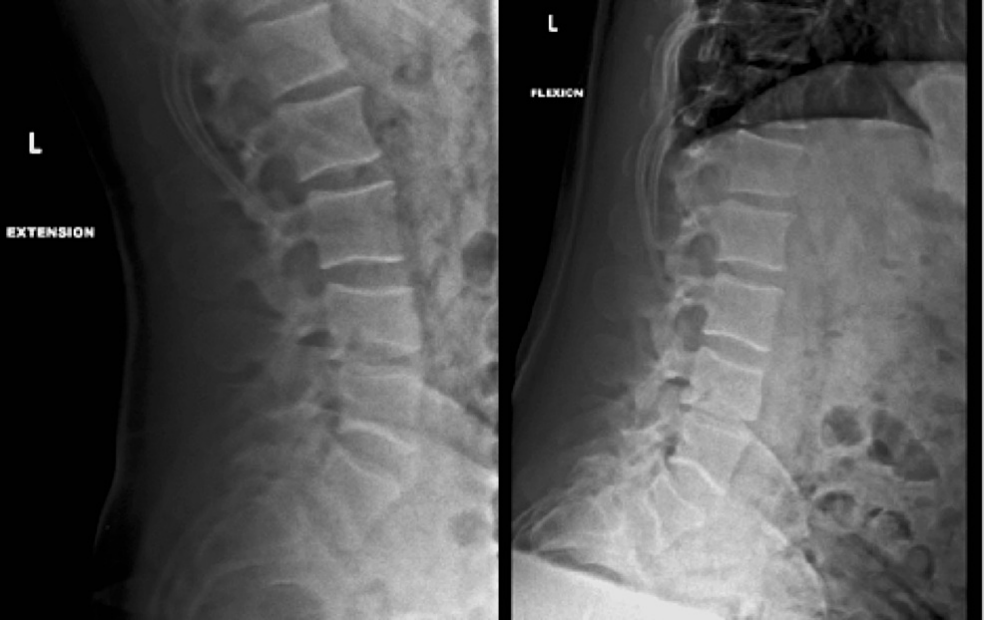
Follow-up flexion and extension X-rays 4 months post-injury demonstrating no mechanical instability

A 33-year-old male presented due to a motor vehicle accident. At presentation, he was hemodynamically stable. Neurological examination revealed central and paraspinal tenderness to palpation in the lumbar and sacral regions with 5/5 strength, 2+ reflexes, and intact sensation in the upper extremities and left lower extremity. The right lower extremity was unable to be tested due to a distal tibia-fibular fracture with soft-tissue swelling, and evidence of comminuted fibular fracture. CT of the head, neck, and cervical spine showed no acute processes. CT of the lumbar region revealed an L4 burst fracture with 3 mm of retropulsion, minimal kyphosis, 20% vertebral body loss of height, and no significant canal narrowing (Figure 1). MRI of the lumbar spine re-demonstrated L4 burst fracture and showed vertebral height loss that was unchanged from initial presentation, with widening of the facet joints, left greater than right, and edema, reflecting definitive PLC injury (Figures 2A, 2B). The patient’s TLICS score was determined to be 5 (2,0,3). The patient was offered surgical stabilization but refused. Upright X-rays of the thoracolumbar did not demonstrate the progression of kyphotic deformity with axial loading. The patient was given a lumbar-sacral orthosis (LSO) brace and was instructed to wear it at all times. At 1 week and 3-month follow-ups, the patient had minimal back pain and the patient remained neurologically intact. At the 4-month follow-up, the patient was still using the brace but was able to return back to regular daily activities and there was no evidence of changes clinically or radiographically (Figure 3).

## Discussion

It is often reasonable to pursue operative or nonoperative management for patients with burst fractures that are neurologically intact. Surgical stabilization can provide better short-term pain relief and sooner return to work at the expense of cost to the patient and the healthcare system [8,9]. The reduction in pain with surgical stabilization is lost over time and there does not seem to be a significant difference at 5-year follow-up in terms of school, work, pain, and quality of life. [1]. There are no defined criteria for further imaging with MRI and it is up to the surgeon to decide whether MRI is necessary to assess the ligamentous injury, disc herniation, and vertebral body edema. However, even when an MRI is obtained, it is common for patients without evidence of PLC injury and therefore, a TLICS score (Table 1) of 2, to still require surgery for persistent back pain or progressive kyphosis [6].

**Table 1:** TLICS system The TLICS system proposed by Patel et al. [3] TLICS: thoracolumbar injury classification and severity

Type of Fracture		Neurological Status		Integrity of Posterior Ligamentous Complex	
Compression	1	Intact	0	Intact	0
Burst	2	Nerve root injury	2	Suspected or intermediate	2
Translation/Rotation	3	Complete cord injury	2	Definite injury	3
Distraction	4	Incomplete cord injury	3		

Tan et al. performed a meta-analysis of neurologically intact patients with burst fractures of T10-L5 without evidence of PLC injury, i.e., TLICS score of 2 that failed conservative management. Seventy-eight of 601 (13%) failed conservative treatment requiring surgical stabilization [6]. Seven of the studies with 378 patients included in the analysis reported specific reasons for failure. Most patients failed due to either back pain (28 patients), kyphosis (19 patients), or both (1 patient). Only five of the 378 (1.3%) patients failed due to neurologic deficit, two for new radicular pain requiring decompression and fusion, and three for unspecified neurologic deficit [6].

Risk factors such as kyphotic angle, interpedicular distance, and visual analog scale have been identified for failure of conservative management in patients with TLICS score ≤ 3, but no prognostic indicators for successful conservative management in patients with TLICS ≥ 5 have been identified in the literature [2,6]. Vaccaro et al. created the TLICS score with three categories of instability: 1) immediate instability indicated by the morphology of injury, 2) long-term instability indicated by PLC injury, and 3) neurologic stability indicated by neurologic exam [4]. Unfortunately, there is no data or studies that evaluate patients with burst fractures and PLC injuries that are managed conservatively to evaluate long-term instability. There is only one other case report of a patient with a TLICS score of 5 being managed conservatively [5].

We demonstrate successful conservative management of a burst fracture patient with TLICS of 5. We believe that there is a certain subset of patients with thoracolumbar burst fractures that are neurologically intact with PLC injury that can be managed conservatively. This requires further research to determine which patients would fall in this cohort.

## Conclusions

With the presentation and outcome of our patient, we believe that certain subsets of patients with thoracolumbar burst fractures can be managed conservatively. These patients are ones that are neurologically intact with PLC injuries. There is a necessity for further investigation into other subsets of patients that may also be managed conservatively but with an operative TLCIS score. This helps to demonstrate that although the TLICS score is very useful, there may be situations where it does not offer the best management for spinal fracture patients.
